# Unilateral Hypertrophy of the Tensor Fasciae Latae Muscle: A Case Report

**DOI:** 10.7759/cureus.58547

**Published:** 2024-04-18

**Authors:** Jose Daniel Chiriboga Arosemena, Esteban Holguin

**Affiliations:** 1 Orthopaedics and Traumatology, Qra Medicina Especializada, Quito, ECU

**Keywords:** tensor fasciae latae (tfl), tumor imaging, benign lesion, pseudotumor, soft-tissue tumor, muscular hypertrophy

## Abstract

Unilateral hypertrophy of the Tensor Fasciae Latae (TFL) muscle is a rare condition often characterized by a palpable mass in the lower limbs or hip pain. Despite its rarity, several causative factors have been identified, necessitating accurate diagnosis and appropriate management. Here, we present the case of a 53-year-old patient who sought outpatient consultation for a mass in the anterolateral aspect of the right thigh. Through this case study, we aim to contribute to the limited literature on this condition by discussing our diagnostic approach, management plan, and outcomes. Upon presentation, the patient underwent a thorough physical examination, revealing a non-tender, sessile mass seemingly originating in the deep connective tissue of the thigh. A magnetic resonance image (MRI) was performed to confirm the diagnosis and assess the extent of muscle involvement. This noninvasive modality provided valuable insights into the nature and localization of the mass, providing the diagnosis and guiding subsequent management decisions. Given the benign nature of the condition and absence of associated symptoms, conservative management was favored. Physical therapy focusing on stretching and strengthening exercises was initiated to address the underlying probable causes and improve functional capacity. Close monitoring through regular follow-up appointments was also recommended to track the progression of the hypertrophy and ensure symptomatic relief. Unilateral hypertrophy of the TFL muscle is a rare entity that presents diagnostic and management challenges. Through our case study, we have highlighted the importance of a comprehensive diagnostic workup, including imaging studies, in confirming the diagnosis and guiding management decisions. Conservative approaches, such as physical therapy, can effectively manage symptoms and improve quality of life in affected individuals. Continued research and documentation of cases are essential to expand our understanding of this condition and refine treatment strategies.

## Introduction

Palpable masses in the lower limbs constitute a common concern prompting patients to seek medical consultation [1]. The differential diagnoses for such masses are extensive, encompassing neoplastic diseases, both benign and malignant, pseudotumors, selective muscle or muscle group hypertrophies, and fluid collections, among other possibilities [2,3]. Soft tissue pseudotumors, although rare, pose diagnostic challenges with limited literature available [4]. While isolated or asymmetric muscle hypertrophies have been recognized as a prevalent cause of soft tissue pseudotumors, Tensor Fasciae Latae (TFL) hypertrophy has been infrequently documented, and to our knowledge, does not have a precise incidence or prevalence rate in the general population [1,4-6]. Previous studies have associated TFL hypertrophy with conditions such as partial ruptures of the gluteus medius and minimus tendons, complications post-total hip replacement (THR), and lumbar radiculopathy [6-9]. Ilaslan et al. presented a series of eight cases of hypertrophy of the tensor fasciae latae (HTFL), revealing associations with relevant medical histories, including THR, periacetabular osteotomy, trauma, melanoma, anticoagulation, and diabetic peripheral neuropathy [4]. Notably, two patients in the series lacked significant medical history. Despite these associations, the exact etiology remains undetermined, with a prevailing theory suggesting overload in the coxofemoral joint secondary to gait disturbance linked to the aforementioned nosological entities [1].

Patients typically seek consultation due to palpable masses on the lateral or anterolateral aspect of the thigh that may be accompanied by unilateral (ipsilateral) thigh pain [4]. The preferred diagnostic modalities for HTFL are computed axial tomography (CT) or magnetic resonance imaging (MRI) due to their ability to reveal characteristic features, allowing physicians to circumvent invasive procedures that may not yield additional insights compared to imaging alone. MRI images distinctly portray TFL enlargement compared to the contralateral muscle, alongside gluteal hypotrophy [2,5]. This text aims to share experiences and available evidence related to the diagnosis and management of this pathology.

## Case presentation

**Figure 1:**
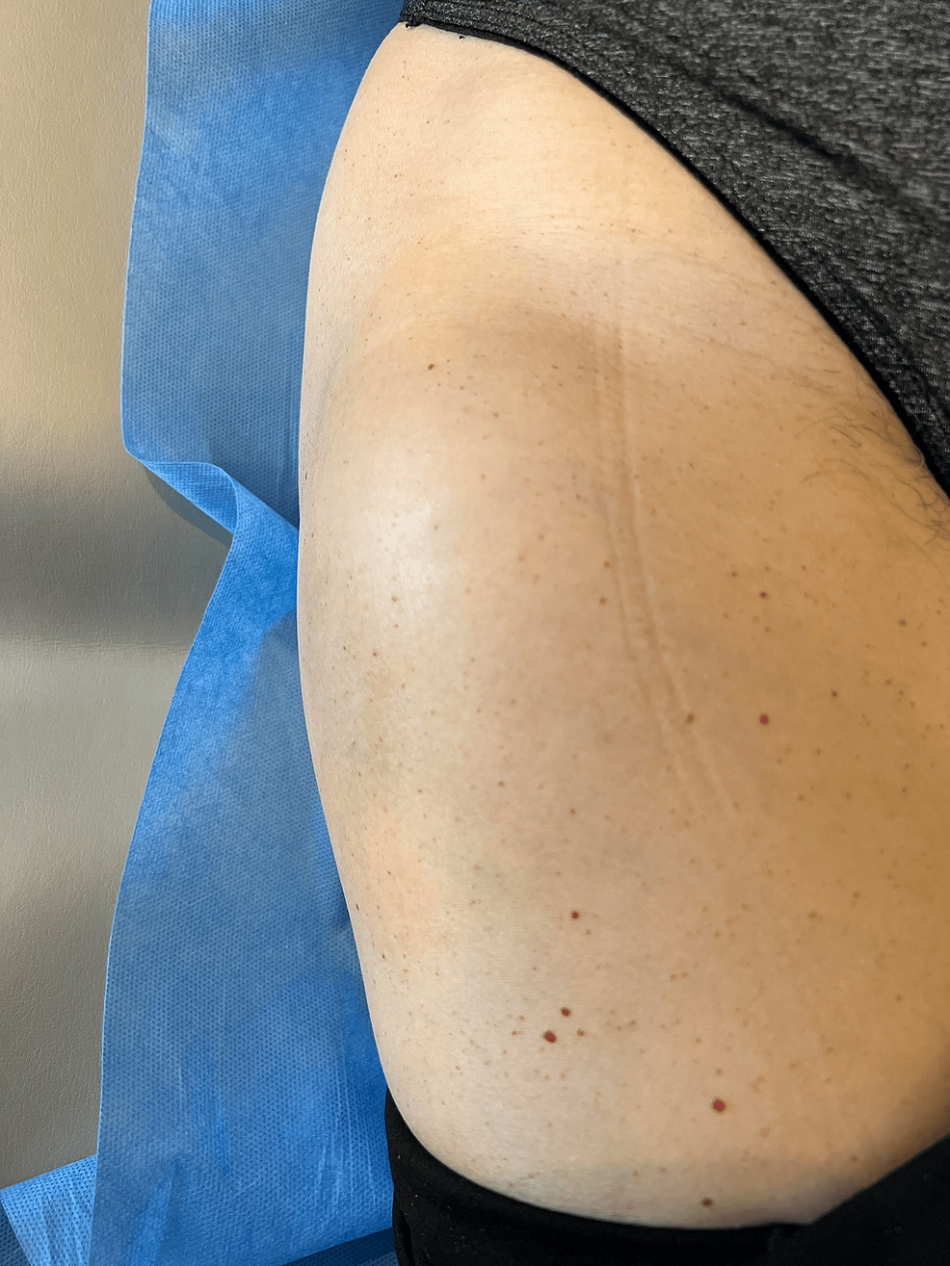
Photograph of the lower right limb and mass Picture taken of the patient while lying in a supine position with the hip in neutral position.

This is a 53-year-old female patient, with no relevant personal history, who sought care in consultation with a specialist in orthopedics and traumatology because she had a tumor on the anterolateral aspect of the right thigh, in the proximal third. She had first noticed it approximately 3 months before the consultation, and it had been of insidious growth and was occasionally accompanied by ipsilateral groin pain. On physical examination, a subcutaneous mass could be seen, and on palpation, it had a diameter of approximately 5 cm, sessile, non-painful, and soft in consistency (Figure 1).

**Figure 2:**
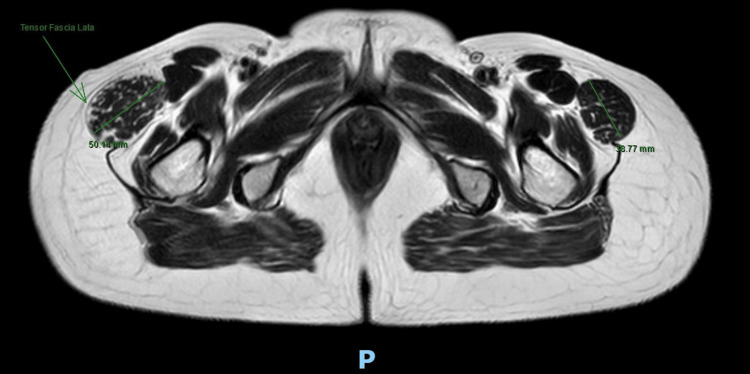
T1-Weighted MRI: Axial cut Hypertrophied tensor fasciae latae muscle (TFLM) (arrow), and transverse measurements of both TFLM (50.14 mm Right and 38.77 mm Left).

**Figure 3:**
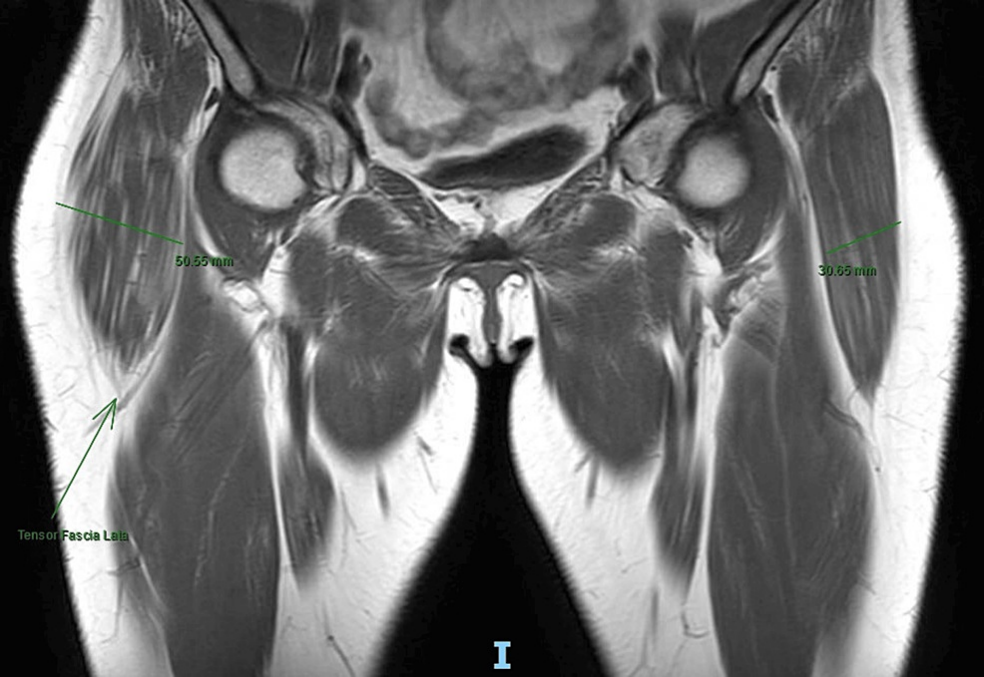
T1-Weighted MRI: Coronal cut Hypertrophied TFL (arrow) and both TFL measurements (50.55 mm Right and 30.65 mm Left). TFL: tensor fasciae latae

**Figure 4:**
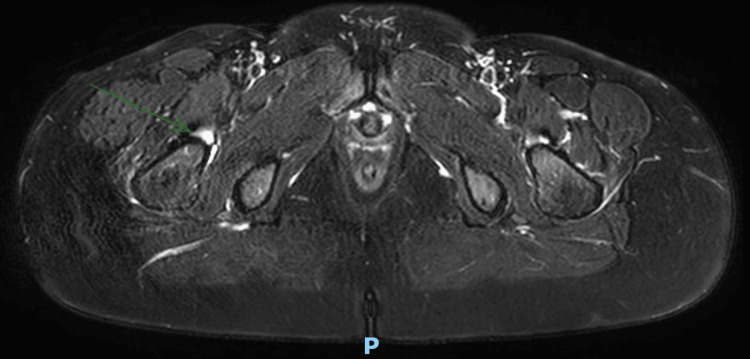
T2-Weighted MRI: Axial cut Gluteus medius enthesopathy (arrow).

**Figure 5:**
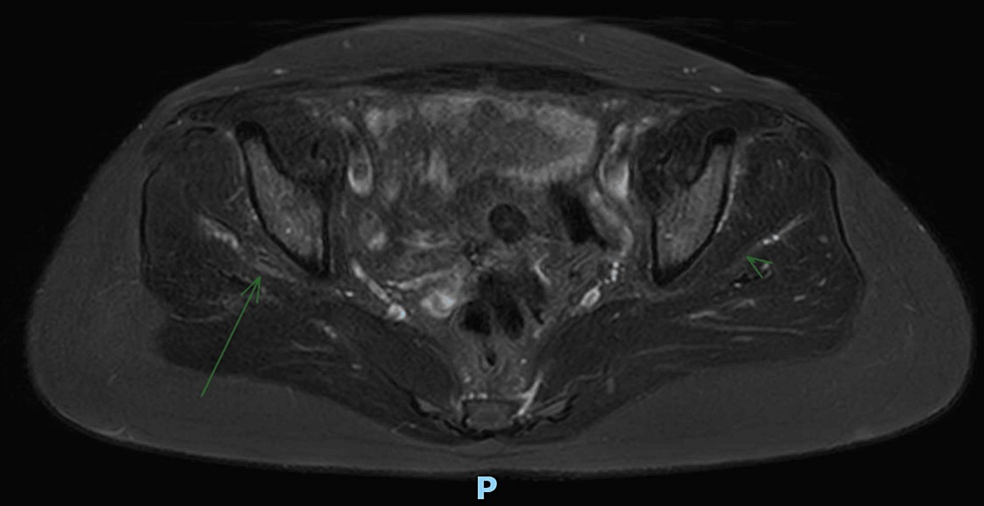
T2-Weighted MRI: Axial cut Ipsilateral hypertrophy of the gluteus medius (large arrow) and contralateral atrophy of the gluteus medius (small arrow).

**Figure 6:**
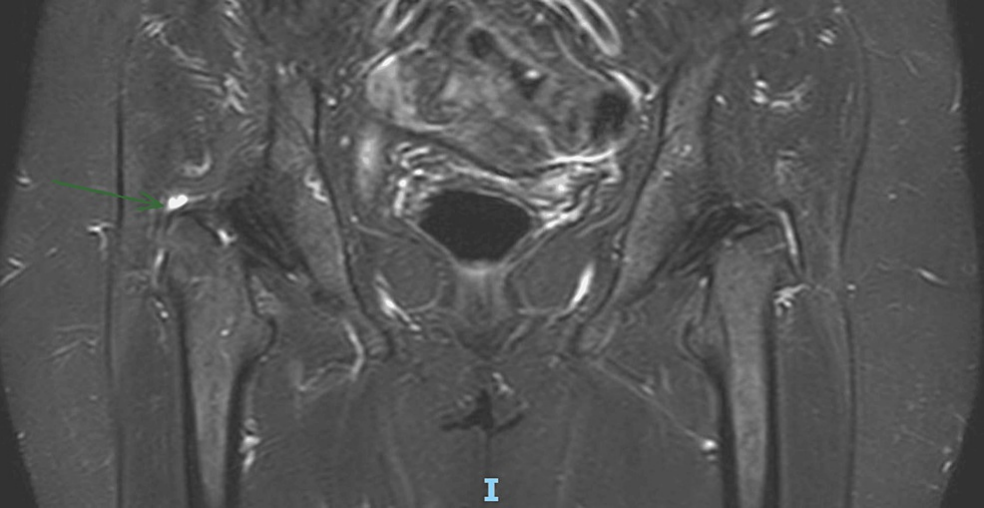
STIR Sequence: Coronal view The right-sided enthesopathy is signaled with an arrow. STIR: short tau inversion recovery

Imaging studies (MRI) were performed, and these demonstrated hypertrophy of the TFL with increased thickness of the muscle fibers, as well as heterogeneous and striated infiltration of hyperintense tissue in between the muscle fibers in the T1 sequence. It also shows a diameter of 50 mm at the muscle belly of the affected TFL compared to the 38 mm diameter seen in the contralateral TFL (Figures 2-3). It also became evident, in the T2 and short tau inversion recovery (STIR) sequences, that there is an enthesopathy at the insertion of the ipsilateral gluteus medius muscle, as well as hypotrophy of the same muscle, contralateral to the hypertrophic TFLM (Figures 4-6).

The patient had no known allergies and was treated initially with a short course of diclofenac, administered orally at 25 mg twice a day, coupled with tramadol at the same oral dosage for three days. Simultaneously, the patient was referred to our rehabilitation center to commence a comprehensive therapeutic regimen. There, she underwent a multidimensional approach, encompassing sedative therapy, force conditioning, range of motion exercises, and proprioception therapy, all scheduled three times a week. During subsequent follow-up consultations, the patient reported a substantial alleviation of the presenting pain, with manifestations limited to supervised exertion exercises. The initial follow-up occurred 10 days post-diagnosis and commencement of the treatment plan. At the second follow-up (30 days), the patient communicated experiencing low-intensity, sporadic, stinging pain in her right groin, which, reassuringly, had minimal repercussions on her daily life. The mass, however, had not diminished in size, apparently. In light of the evolving clinical picture, the physiotherapy sessions were extended, totaling 12 sessions to ensure sustained rehabilitation progress.

## Discussion

HTFL stands as a rare nosological entity. To such an extent that the existing literature consists almost exclusively of case reports and lacks prevalence and incidence reports [1, 3-7]. It is necessary to carry out a meticulous approach to diagnosis through a comprehensive review of past medical history and physical examination. Given its infrequency [1, 3-7], practitioners should exercise prudence in directing diagnostic suspicion and confirmatory measures, prioritizing noninvasive approaches to avoid unnecessary procedures and their associated risks [5]. The uniqueness of HTFL within the spectrum of soft tissue pseudotumors underscores the importance of differing the realization of invasive tests like biopsies, until after imaging exams have been realized. The limited utility of histological studies in influencing treatment decisions or success rates deems such procedures unwarranted, particularly when weighed against the inherent risks of infection, bleeding, pain, and psychological distress imposed on the patient. Such studies would be appropriate if the imaging study presented signs suggestive of malignant neoplastic disease, or when the nature of the mass cannot be accurately determined through indirect methods [1, 2].

In the context of HTFL, imaging studies emerge as the cornerstone for an accurate diagnosis, with CT and MRI being the preferred modalities, MRI is preferred over CT due to the fact that it does not subject the patient to ionizing radiation and that it shows more clearly the presence or absence of tendinopathies and enthesopathies which have been linked to this disease [2, 4]. These noninvasive approaches provide crucial insights into the distinctive features of HTFL, including the enlargement of the TFL compared to the contralateral muscle and associated gluteal lesions, if present, as well as the absence of characteristics corresponding to other lesions like cysts, arterial-venous malformations, or tissue invasion which can be seen in other diseases that comprise the differential diagnoses for a mass of the lower extremities [3, 10]. Such diagnostic clarity allows physicians to formulate effective management strategies without subjecting patients to the unnecessary perils associated with invasive procedures like biopsies or even unwarranted surgeries. The emphasis on imaging techniques aligns with the broader paradigm shift toward patient-centric care, safeguarding against unjustified harm while striving for precise and efficacious diagnoses [5].

Furthermore, the clinical presentation of patients with HTFL typically involves palpable masses on the lateral or anterolateral aspect of the thigh, often accompanied by unilateral thigh pain. This symptomatology should nudge the direction of the diagnostic focus, guiding healthcare professionals to consider HTFL in their differential diagnoses [4]. The differential diagnoses for this disease can be separated into four groups: neoplastic diseases, benign lesions, lymphadenopathy, and HTFL [10]. Within the first group, there have been reports of primary and secondary lesions of this nature, and should always be considered [10]. Within the second group, we can find fluid collections like the Morel-Lavallèe lesion, abscesses, benign vascular tumors, and lipomas. The third group consists of lymphadenopathies which constitute a rather common finding in the clinical setting [10, 11]. The recognition of the aforementioned clinical cues, coupled with a judicious use of imaging studies, ensures a streamlined and patient-friendly diagnostic process. Moreover, the awareness of HTFL's associations with conditions such as partial ruptures of the gluteus medius and minimus tendons, complications post-THR, and lumbar radiculopathy serves as valuable knowledge in refining the diagnostic suspicion, steering healthcare practitioners toward a more accurate evaluation [6-9].

Importantly, while the concern for a malignant tumor may weigh on the minds of healthcare providers, the decision to prioritize imaging studies does not significantly prolong the diagnostic timeline [3]. On the contrary, it expedites the identification of HTFL, allowing for swift initiation of appropriate management. In doing so, the approach minimizes the patient's exposure to unnecessary invasive procedures, underscoring the paramount importance of patient well-being in the diagnostic process. By promptly employing imaging techniques, healthcare professionals can efficiently navigate the diagnostic landscape, balancing the imperative to rule out malignancy with the commitment to a patient-centered and evidence-based diagnostic pathway [8, 9].

## Conclusions

In conclusion, the case of TFL hypertrophy presented here highlights the complexity and rarity of this pathology. Although the literature on soft tissue pseudotumor remains limited, the characteristics of TFL hypertrophy as a causal factor further complicate its diagnosis and treatment. The interaction between muscular components and compensatory mechanisms, as demonstrated in this case, highlights the importance of a comprehensive approach in both the history and physical examination for an accurate diagnostic suspicion.

Additionally, the text emphasizes the judicious use of diagnostic imaging techniques, particularly CT or MRI. This is because these techniques provide valuable information about the characteristics of TFL hypertrophy and help avoid unnecessary invasive procedures such as biopsies. With careful awareness of the potential risks associated with invasive procedures, the success and risks of future research into this disease are carefully weighed. This cautious approach is in line with the spirit of patient-centered care, which prioritizes avoiding unjustified harm while aiming for an accurate diagnosis.

As demonstrated in this case, patient management and follow-up play a vital role in the overall care strategy. Referral to physical rehabilitation to address underlying muscle weakness and prescribing non-steroidal anti-inflammatory drugs (NSAIDs) for pain management reflect a holistic approach to patient health. Clear and accessible communication with patients, using simple language and identifiable analogies, emerges as an important aspect of promoting patient understanding and compliance with recommended treatment plans. Ultimately, this case represents a valuable contribution to the medical understanding of TFL hypertrophy, encouraging clinicians to be diligent in its diagnosis, adopt conservative management strategies, and prioritize patient safety throughout the investigation and treatment process.
